# South African amaXhosa patients with atopic dermatitis have decreased levels of filaggrin breakdown products but no loss-of-function mutations in filaggrin^☆^

**DOI:** 10.1016/j.jaci.2013.09.053

**Published:** 2014-01

**Authors:** Fatemah Thawer-Esmail, Ivone Jakasa, Gail Todd, Yaran Wen, Sara J. Brown, Karin Kroboth, Linda E. Campbell, Grainne M. O'Regan, W.H. Irwin McLean, Alan D. Irvine, Sanja Kezic, Aileen Sandilands

**Affiliations:** a Division of Dermatology, University of Cape Town, Cape Town, South Africa; b Faculty of Food Technology and Biotechnology, Department of Chemistry and Biochemistry, Laboratory for Analytical Chemistry, University of Zagreb, Zagreb, Croatia; c Department of Dermatology and Genetic Medicine, University of Dundee, Dundee, United Kingdom; d Department of Paediatric Dermatology, Our Lady's Children's Hospital Crumlin, Dublin, Ireland; e National Children's Research Centre, Our Lady's Children's Hospital Crumlin, Dublin, Ireland; f Department of Clinical Medicine, Trinity College, Dublin, Ireland; g Coronel Institute of Occupational Health, Academic Medical Center, Amsterdam, The Netherlands

To the Editor:

Loss-of-function (LOF) mutations in the filaggrin gene (*FLG*) are the strongest known genetic risk factors for atopic dermatitis (AD). The genetic architecture of *FLG* mutations is well established in European, Japanese, and selected Chinese populations, but their contribution to AD in African populations is not well understood. The only data on *FLG* mutations in Africans come from a recent study conducted in Ethiopia^1^ that studied 103 patients with AD, 7 patients with ichthyosis vulgaris (IV), and 103 healthy controls. This study identified only a single novel mutation (a 2-bp deletion, 632del2), by direct sequencing of *FLG* in a patient with AD.

To investigate the role of filaggrin in the etiology of AD in South Africa, we studied 69 children with AD from the amaXhosa community along with 81 age-, ethnic- and sex-matched controls, with no history of AD. The patients (n = 69) and controls (n = 81) were recruited from tertiary referral AD clinics in Cape Town. Clinical and demographic characteristics of control subjects and patients with AD are outlined in Table I. The study was conducted in accordance with the Helsinki Declaration and was approved by the Human Research Ethics Committee of the Faculty of Health Sciences of the University of Cape Town. Written consent/ascent in the amaXhosa language was obtained from the patients or their parents.

**Table I:** Clinical and demographic characteristics of control subjects and patients with AD

	N	Age (y), median (range)	Male gender, n (%)	NESS, median (range)	IgE n, median (range)	Ichthyosis	Perifollicular hyperkeratosis	Palmar hyper linearity
CTRL	81	5 (1-31)	43 (53)	—	—	—	—	—
Patients with AD	69	9 (4-29)	36 (52)	10 (4-15)	57, 2114 (12-10500)	68 of 69	21 of 69	60 of 69

*CTRL*, Healthy controls; *NESS*, Nottingham Eczema Severity Score.

*AD was diagnosed by an experienced dermatologist by using the UK diagnostic criteria.

The entire coding sequence of the *FLG* gene was directly sequenced (as described previously^2^) in 31 patients with AD with prominent features of IV, that is, those who should most likely have *FLG* mutations. Sequencing of PCR products was performed by a core facility (DNA Sequencing and Services, University of Dundee, Dundee, United Kingdom) according to our standard operating procedures. The entire collection was additionally typed for the previously known *FLG* mutations R501X, 2282del4, R2447X, and S3247X by using custom-made Taqman allelic discrimination assays.^2^ Although the primers used for the amplification of the *FLG* gene were originally optimized for the sequencing of European populations,^2^ in the 31 amaXhosa patients with AD who were sequenced, we identified 124 mutations (synonymous and nonsynonymous) throughout exon 3 of the *FLG* gene by using these methods. Identification of these silent and nonpathogenic missense mutations in the *FLG* gene indicates minimal allele dropout using these primer sets. None of these identified mutations was predicted to lead to loss of filaggrin at the protein level. Screening of the entire collection of patients for the *FLG* mutations R501X, 2282del4, R2447X, and S3247X showed that all samples were wild type for these mutations.

In addition to gene sequencing, in all patients with AD and controls, we determined the concentrations of filaggrin breakdown products in the stratum corneum (SC). Filaggrin is degraded in the later stages of epidermal differentiation into free amino acids (FAA) and their derivatives; a major proportion of the total SC FAA (70% to 100%) is derived from filaggrin.^3^ The most common amino acid residues in filaggrin repeats are basic amino acids such as histidine (413 of 4061 residues; 10.17%) and arginine (440 of 4061 residues; 10.83%) and the polar residue glutamine (367 of 4061; 9.04%) (see Fig E1 in this article's Online Repository at www.jacionline.org). Histidine is enzymatically deaminated to trans-urocanic acid (trans-UCA).^3^ Trans-UCA, which is converted to cis-UCA on ultraviolet irradiation, functions as a major chromophore and exerts immunomodulatory effects in the skin.^3^, ^4^ UCA maintains an acidic pH in the skin, which is crucial for the optimal function of several enzymes in the SC and antimicrobial defence.^3^, ^4^ Another abundant amino acid glutamine is further converted into pyrrolidone-5-carboxylic acid (PCA). PCA is highly hygroscopic and is one of the major components of the natural moisturizing factor, thus providing a humectant effect by retaining water in the SC.^3^, ^4^ Filaggrin degradation products thus have multiple functions.

*FLG* mutations lead to reduced levels of filaggrin degradation products in the SC in a dose-dependent fashion.^5^ It has been shown that moderate-to-severe AD also has an effect on SC filaggrin expression^6^ as well as on the levels of filaggrin degradation products^5^ possibly due to the systemic T_H_2 immune response.^6^

In the present study, the levels of both PCA and UCA and their sum were significantly decreased in the SC of patients with AD than in control subjects (Fig 1; see Table E1 in this article's Online Repository at www.jacionline.org). The magnitude of reduction between cases and controls in this study was approximately 20%. Similar results were obtained when comparing total FAA content as well as total FAA including their derivatives, PCA, UCA, citrulline, and ornithine (Fig 1; Table E1). While *FLG* mutations are the major determinants of the level of filaggrin breakdown products in the SC, it has been previously shown that their levels are significantly reduced in European populations both in nonlesional skin of patients with AD with *FLG* mutations and in patients without *FLG* mutations.^5^ In this study, we have replicated these filaggrin breakdown product findings in patients with AD without *FLG* mutations in an African population. This is consistent with the *in vitro* findings of Howell et al^6^ and Pellerin et al^7^ and highlights the fact that there is an interplay between the skin barrier and a systemic immunologic process, with systemic T_H_2 inflammation causing a decrease in SC filaggrin expression.

**Fig 1:**
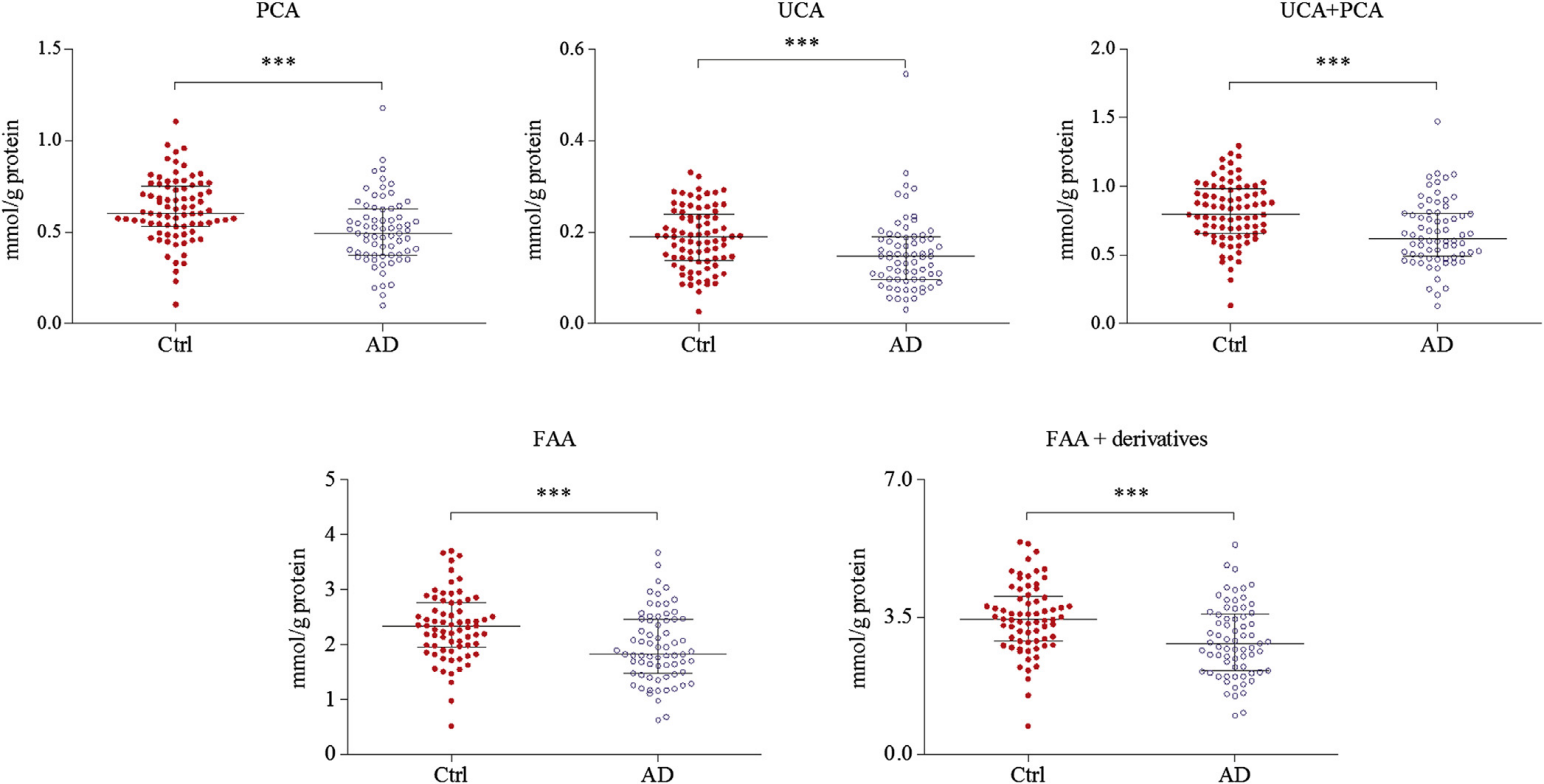
Levels of filaggrin degradation products in the SC of healthy controls (Ctrl, n = 81) and patients with AD (n = 69) (median with interquartiles). *FAA + derivatives*, FAA including UCA, PCA, citrulline, and ornithine. ***P < .001 as determined by the 2-tailed Wilcoxon-Mann-Whitney test.

The SC profiles of filaggrin breakdown products are highly informative in this African population because they provide a second look, in addition to direct sequencing, for *FLG* mutations. In our study, we demonstrate that the diminution of filaggrin breakdown products is consistent with what we have seen in European patients with AD who are wild type for *FLG* mutations. The magnitude of this reduction is much less than that seen in patients with AD with *FLG* mutations, even in African patients with clinical findings consistent with IV who could be expected to have *FLG* mutations. In Europeans with a single *FLG* LOF mutation, expression of these products is reduced by approximately 50%.^5^ We here provide strong and complementary evidence for an absence of *FLG* mutations in this African population through 2 analytical methodologies. Given that *FLG* mutations are well established as the strongest and most important risk factor for AD in European, Japanese, and Chinese populations, this work clearly implies significant genetic heterogeneity between AD in the African population and AD in these populations. The genetics of AD is not well studied in African populations; in addition to the Ethiopian study referenced earlier, 2 studies in African American populations have found low frequencies of European-derived *FLG* mutations,^8^, ^9^ presumably due to European population admixture.^10^ While it is tempting to speculate, on the basis of this work, that there could be a major (non-*FLG*) African-specific gene for IV and/or AD, African populations will require specific studies and dedicated collections to disclose African population-specific major genetic risks for AD.

In conclusion, *FLG* LOF mutations are not a significant contributor to AD in the amaXhosa population. When combined with previous findings in the Ethiopian population, the contribution of these mutations to AD in Africans seems to be at great variance with their major role in European and Asian populations. Further work is required in African populations to better understand the genetic basis of AD in these populations.

## Figures and Tables

**Fig E1:**
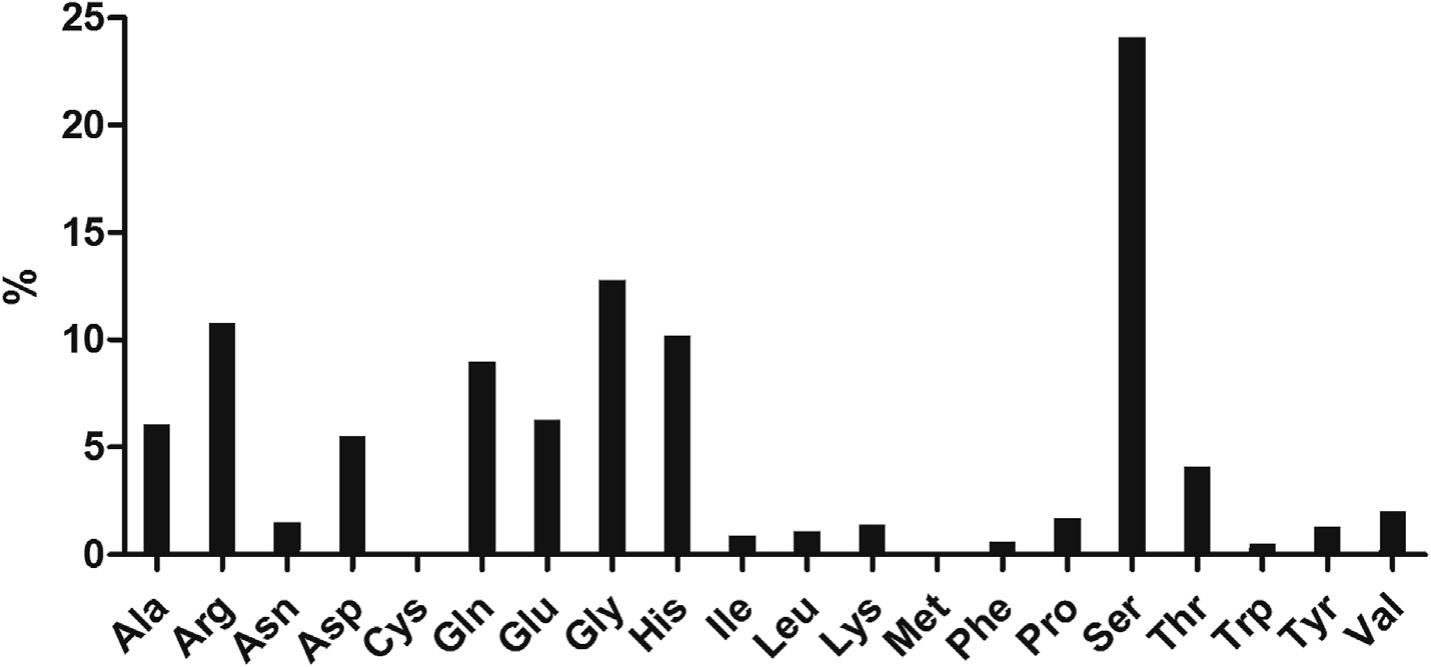
Amino acid composition of filaggrin. *Ala*, Alanine; *Arg*, arginine; *Asn*, asparagine; *Asp*, aspartate; *Cys*, cysteine; *Gln*, glutamine; *Glu*, glutamate; *Gly*, glycine; *His*, histidine; *Ile*, isoleucine; *Leu*, leucine; *Lys*, lysine; *Met*, methionine; *Phe*, phenylalanine; *Pro*, proline; *Ser*, serine; *Thr*, threonine; *Trp*, tryptophan; *Tyr*, tyrosine; *Val*, valine.

**Table E1:** The levels of filaggrin degradation products and differences between healthy controls and patients with AD with corresponding 95% CIs

Filaggrin degradation product∗ (mmol/g protein)	Median (range)		Difference (95% CI)	*P* value
	CTRL	AD		
PCA	0.60 (0.11-1.10)	0.49 (0.10-1.18)	−0.11 (−0.181 to −0.068)	<.0001
UCA	0.19 (0.03-0.33)	0.15 (0.03-0.55)	−0.043 (−0.063 to −0.017)	.0006
FAA	2.32 (0.51-3.70)	1.82 (0.62-3.67)	−0.51 (−0.60 to −0.18)	.0005
FAA + derivatives	3.44 (0.72-5.40)	2.82 (0.98-5.34)	−0.62 (−0.91 to −0.29)	.0002

∗ PCA and UCA have been determined by HPLC from the SC samples collected by adhesive tape strips as described elsewhere (Dapic I, Jakasa I, Yau N, Kezic S, Kammeyer A. Evaluation of an HPLC method for the determination of natural moisturizing factors in the human stratum corneum. Anal Lett 2013;46:2134-44.) FAA and their derivatives from the SC tapes were analyzed by using ultra performance liquid chromatography-tandem mass spectrometry. The concentrations are expressed as mmol of each component/g of protein to compensate for variable harvest of the SC.

## References

[1] Winge M.C., Bilcha K.D., Liedén A., Shibeshi D., Sandilands A., Wahlgren C.-F. (2011). Novel filaggrin mutation but no other loss-of-function variants found in Ethiopian patients with atopic dermatitis. Br J Dermatol.

[2] Sandilands A., Terron-Kwiatkowski A., Hull P.R., O'Regan G.M., Clayton T.H., Watson R.M. (2007). Comprehensive analysis of the gene encoding filaggrin uncovers prevalent and rare mutations in ichthyosis vulgaris and atopic eczema. Nat Genet.

[3] Harding C.R., Aho S., Bosko C.A. (2013). Filaggrin - revisited. Int J Cosmet Sci.

[4] McAleer M.A., Irvine A.D. (2013). The multifunctional role of filaggrin in allergic skin disease. J Allergy Clin Immunol.

[5] Kezic S., O'Regan G.M., Yau N., Sandilands A., Chen H., Campbell L.E. (2011). Levels of filaggrin degradation products are influenced by both filaggrin genotype and atopic dermatitis severity. Allergy.

[6] Howell M.D., Kim B.E., Gao P., Grant A.V., Boguniewicz M., DeBenedetto A. (2007). Cytokine modulation of atopic dermatitis filaggrin skin expression. J Allergy Clin Immunol.

[7] Pellerin L., Henry J., Hsu C.-Y., Balica S., Jean-Decoster C., Méchin M.-C. (2013). Defects of filaggrin-like proteins in both lesional and nonlesional atopic skin. J Allergy Clin Immunol.

[8] Gao P.-S., Rafaels N.M., Hand T., Murray T., Boguniewicz M., Hata T. (2009). Filaggrin mutations that confer risk of atopic dermatitis confer greater risk for eczema herpeticum. J Allergy Clin Immunol.

[9] Margolis D.J., Apter A.J., Gupta J., Hoffstad O., Papadopoulos M., Campbell L.E. (2012). The persistence of atopic dermatitis and filaggrin (FLG) mutations in a US longitudinal cohort. J Allergy Clin Immunol.

[10] Kidd J.M., Gravel S., Byrnes J., Moreno-Estrada A., Musharoff S., Bryc K. (2012). Population genetic inference from personal genome data: impact of ancestry and admixture on human genomic variation. Am J Hum Genet.

